# Analysis of Rehabilitation Effect of Neurology Nursing on Stroke Patients with Diabetes Mellitus and Its Influence on Quality of Life and Negative Emotion Score

**DOI:** 10.1155/2022/1579928

**Published:** 2022-03-10

**Authors:** Yanhong Yang, Guixia Niu, Qing Mi, Feifei Hong, Guiqin Zhang

**Affiliations:** ^1^Second Department of Neurology, People's Hospital Affiliated to Shandong First Medical University, Jinan 271199, Shandong Province, China; ^2^Second Department of Rehabilitation Medicine, People's Hospital Affiliated to Shandong First Medical University, Jinan 271199, Shandong Province, China; ^3^Department of Science Teaching, People's Hospital Affiliated to Shandong First Medical University, Jinan 271199, Shandong Province, China

## Abstract

**Objective:**

To explore and analyze the rehabilitation effect of neurology nursing on stroke patients with diabetes mellitus (DM) and its influence on quality of life and negative emotion score.

**Methods:**

In this experiment, 110 stroke patients with DM diagnosed and treated in our hospital from 2018 to 2020 were randomly selected and assigned to the study group (SG) and the control group (CG) according to different nursing methods, with 55 cases in each group. In SG, they were given neurology nursing. In CG, they were given routine nursing. The rehabilitation efficacy, quality of life, and negative emotion scores were compared between the two groups.

**Results:**

Compared with the CG, the levels of fasting blood glucose, 2 h postprandial blood glucose, and urinary microalbumin in SG were obviously better after treatment. In SG, the proportion of patients with basic recovery and significant improvement after treatment was higher, and the proportion of patients without treatment effect was significantly lower. Overall, the nursing effect of the SG after treatment was better than that of the CG. There was no striking difference in the quality of life and Morisky scores between the two groups before nursing intervention (*P* > 0.05), but the quality of life and Morisky scores of patients in SG were obviously higher than those in CG after nursing intervention. After nursing intervention, SAS and SDS scores of patients in SG were obviously lower than those of patients in CG, and patients in SG were less affected by negative emotions. Questionnaires were used to investigate the satisfaction of patients in both groups, and the results showed that the satisfaction of patients in SG was higher (all *P* < 0.05).

**Conclusion:**

Neurology nursing has better clinical efficacy for stroke patients with DM and has obvious rehabilitation effect. The quality of life and negative emotion score of patients are better, which is worthy of extensive clinical promotion and application.

## 1. Introduction

Cerebral stroke is a cardiovascular and cerebrovascular disease, commonly known as “stroke,” also known as “cerebrovascular accident” (CVA), which is mainly caused by abnormal blood circulation in the brain. It is a sudden and rapid cerebral hemorrhage or cerebral ischemia disease with high morbidity, disability, recurrence, and mortality [1]. A survey shows that stroke has become the first cause of death and the leading cause of disability among Chinese adults in China. DM is a group of metabolic diseases characterized by chronic increase of blood glucose level. Long-term hyperglycemia will lead to dysfunction of various tissues such as the eyes, kidneys, heart, blood vessels, and nerves [2]. DM is the disease with the highest incidence in the world at present. With the social progress and improvement of living standards, the incidence trend is increasing. Some research statistics show that the number of diabetic patients in the world is as high as 25.6% in 2015 [3]. DM is easy to cause various complications, and stroke is a common complication of DM [4]. According to relevant studies, the relative risk of stroke patients with DM is 1.8-6.0, and the combined disability rate and mortality rate are higher [5, 6]. Stroke complicated with DM is quite common in clinic, mostly developing in middle-aged and elderly people. Patients need to take drugs for a long time. Compliance and knowledge of the disease directly affect the blood sugar status and mental health of patients [7, 8]. At present, how to make patients face DM actively and rationally and receive professional and systematic treatment is the most important problem to be solved in a nursing work. If nursing intervention is not carried out in time, it is easy to induce other complications and cause incalculable consequences [9, 10]. Studies have shown that positive intervention should be carried out to further ensure the diagnosis and treatment effect of stroke patients with DM. Neurology nursing is a systematic, comprehensive, and targeted method, which can better meet the patients' physiological and psychological nursing needs. Based on the characteristics of the patient's condition and their own specific situation, they can be given psychological, diet, medication, complications, body and language function exercise, and other aspects of better nursing intervention [11]. This research is aimed at providing references and opinions for future clinical practice by exploring and analyzing the rehabilitation effect of neurology nursing on stroke patients with DM and its influence on the quality of life and negative emotion score. The report is as follows.

## 2. Materials and Methods

### 2.1. Baseline Data

In this experiment, 110 stroke patients with DM diagnosed and treated in our hospital from 2018 to 2020 were randomly selected and assigned to SG and CG according to different nursing methods, with 55 cases in each group. In SG, the ratio of male to female is 28 : 27, the age range is 40-69 years old, the average age is 55.87 ± 5.64 years, and the history of DM is 2-8 years with an average course of 5.13 ± 1.24 years. In CG, the ratio of male to female is 30 : 25, the age range is 43-71 years, the average age is 56.39 ± 5.82 years, and the history of DM is 2-8 years with an average course of 5.51 ± 1.03 years. The two groups are of comparative value (*P* > 0.05, Table 1).

### 2.2. Inclusion and Exclusion Criteria

The inclusion criteria were as follows: (1) patients met the criteria for the diagnosis of stroke and the indications of DM; (2) patients were informed of this research and voluntarily affixed the consent form; and (3) this research has been ratified by the Ethics Committee of our hospital.

The exclusion criteria were as follows: (1) history of drug allergy, (2) liver and kidney dysfunction, and (3) cognitive dysfunction.

### 2.3. Nursing Methods

In CG, patients were given routine nursing intervention, including blood sugar observation, basic nursing, daily ward rounds, and medication according to doctor's advice.

In SG, patients were given neurology nursing intervention, which was mainly divided into four aspects:

1. Basic intervention: medical staff needed to help patients turn over on time and do not squeeze the patient's skin to prevent pressure sores; Medical staff needed to regularly clean up foreign bodies in the respiratory tract to avoid obstructing breathing and pay attention to catheter blockage and slippage. Medical staff needed to help the patient lift his or her lower limbs to prevent venous thrombosis.
2. Psychological care: patients with DM need to take medicine for a long time, and the possibility of recurrence is extremely high, and they worry about the occurrence of stroke disease and do not understand the condition, which will easily cause serious psychological burden to the patients. Therefore, medical staff should communicate with family members and patients more according to the patient's situation, help patients clearly understand their symptoms, establish the source of their negative emotions, and enhance patients' confidence in recovery.
3. Limb rehabilitation nursing: in the early stage, massage rehabilitation was used, and active training was changed according to the patient's recovery condition, slowly improving the patient's activity intensity, carrying out sitting, migration, and other sports, and slowly carrying out the training of standing and walking on a foot walking vehicle. All training intensity is subject to patient tolerance to prevent secondary injury.
4. Language rehabilitation training: according to the patient's own situation and education level, language training should be conducted on time, and listening training should be carried out with radio or TV equipment until the patient can communicate simply.

### 2.4. Scoring Standards

The following indicators were collected on the day of admission and discharge:

1. The blood (200 ml) was taken from the patient's vein on an empty stomach and two hours after a meal, respectively. The changes of blood sugar and the occurrence of adverse diseases in the two groups were observed and compared after treatment. Also, morning urine of patients was taken
2. The Nursing Effect Table developed by our hospital was used to analyze the nursing effect of patients, comprising four levels: basic rehabilitation, obvious improvement, improvement, and ineffectiveness. The total effective rate of each group was calculated according to the number of people in each level
3. The quality of life of patients was assessed by SF-36, which was assigned to eight dimensions: physical health (physiological function, physiological role, physical pain, and general health) and mental health (vitality, social function, emotional role, and mental health). The total score of each dimension was 100 points. The higher the score, the better the patient's quality of life
4. The Morisky Compliance Scale was used to evaluate the treatment compliance of patients before and after nursing intervention from four aspects: medication according to doctor's advice, body quality control, diet control, and appropriate exercise, with a full score of 50 [full compliance: 50, partial compliance: 30-40, and noncompliance: less than 30]
5. Self-rating Anxiety Scale (SAS) and self-rating Depression Scale (SDS) were used to evaluate and compare the psychological states of patients in both groups. A score below 55 is normal, 56-65 is mild anxiety or depression, 66-75 is moderate anxiety or depression, and 76 or above is severe anxiety or depression. The lower the score, the better the psychological state of the patient
6. The Nursing Satisfaction Questionnaire developed by our hospital (including the attitude of medical staff, the efficiency of medical staff, and the explanation of diseases by medical staff) was divided into four options (very satisfied, satisfied, not very satisfied, and dissatisfied) to understand the satisfaction of patients in both groups. Then, the results of the two groups were analyzed to determine which treatment was more effective

### 2.5. Data Analysis

The image was processed by GraphPad Prism 8. SPSS22.0 was used to process the data. A chi-square test and *T*-test were performed on the enumeration data [*n* (%)] and the measuring materials ($\bar{x}\pm s$), respectively. The difference was statistically significant (*P* < 0.05).

## 3. Results

### 3.1. Blood Glucose Level

Compared with the CG (6.93 ± 1.95, 12.79 ± 1.87, and 20.02 ± 5.68), the levels of fasting blood glucose, 2 h postprandial blood glucose, and urinary micro-albumin in SG (6.01 ± 1.02, 11.18 ± 1.21, and 41.65 ± 6.07) were obviously better after treatment. The results showed that the blood glucose level in SG was better (*P* < 0.05) (Table 2).

### 3.2. Nursing Effect

After treatment, the proportion of patients in SG who basically recovered and had obvious improvement (38.18%, 49.09%) was higher than that in CG (20.00%, 30.91%), and the proportion of patients without treatment effect (1.81%) was obviously lower than that in CG (21.82%). Overall, the nursing effect of the SG (98.19%) after treatment was better than that of the CG (78.18) (*P* < 0.05) (Table 3).

### 3.3. Quality of Life

**Figure 1:**
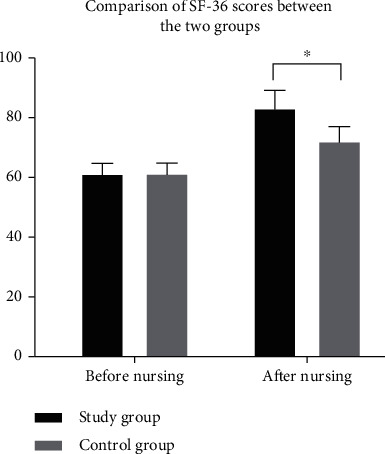
Comparison of SF-36 scores between the two groups before and after nursing intervention. ∗ indicates that there is a difference between the two groups.

There was no significant difference in the SF-36 score between the two groups before nursing intervention (*P* > 0.05). After the intervention, the quality of life score of patients in SG (83.23 ± 5.87) was obviously higher than that in CG (72.14 ± 4.79) (*P* < 0.05) (Figure 1).

### 3.4. Morisky Score

**Figure 2:**
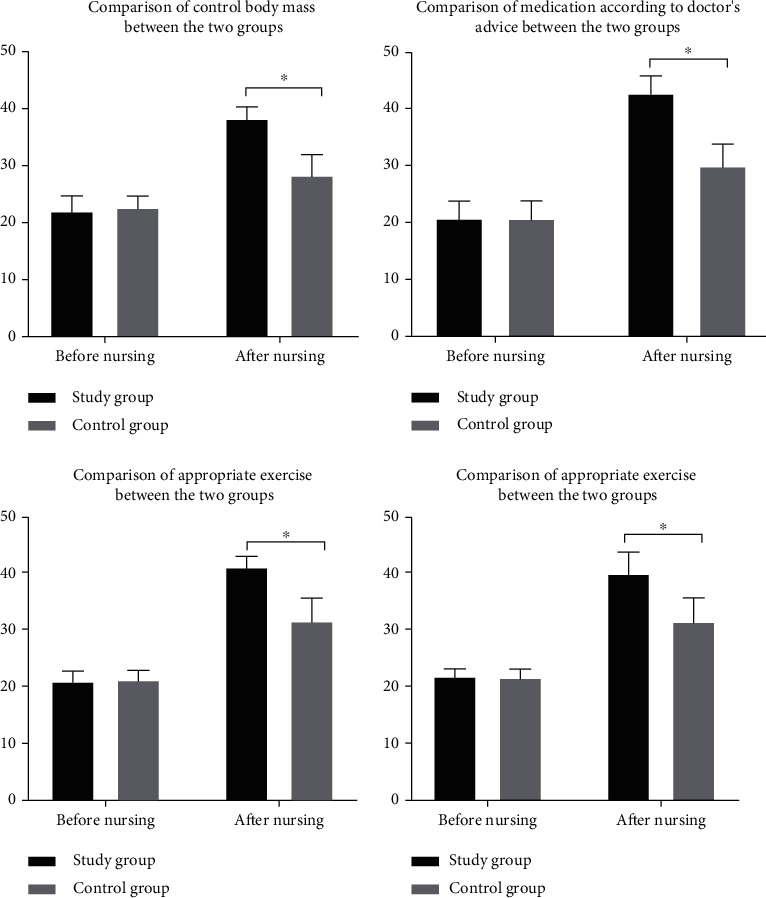
Comparison of Morisky scores between the two groups before and after nursing intervention. ∗ indicates that there is a difference between the two groups.

There was no significant difference in the Morisky score between the two groups before nursing intervention (*P* > 0.05). After the nursing intervention, the patients in SG (38.21 ± 2.14, 42.85 ± 3.02, 41.21 ± 1.84, and 40.07 ± 3.78) were obviously higher than those in CG (28.25 ± 3.68, 30.02 ± 3.87, 31.65 ± 4.17, and 31.56 ± 4.25) in terms of controlling body weight, taking medicine according to doctor's advice, exercising properly and controlling diet (*P* < 0.05) (Figure 2).

### 3.5. Negative Emotion Score

After nursing intervention, SAS and SDS scores of patients in SG (37.12 ± 2.01 and 35.96 ± 2.34) were obviously lower than those of patients in CG (48.49 ± 3.65 and 49.21 ± 3.17), and patients in SG were less affected by negative emotions (*P* < 0.05) (Table 4).

### 3.6. Nursing Satisfaction

**Figure 3:**
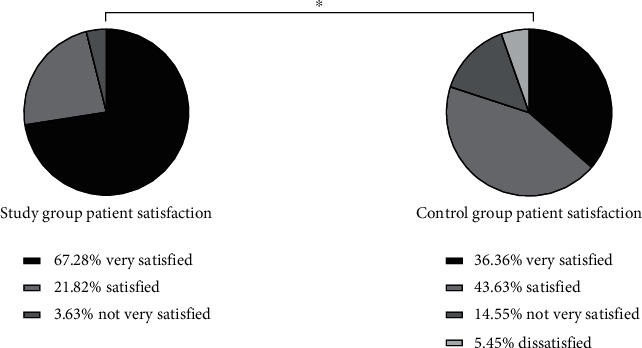
Comparison of SF-36 scores between the two groups before and after nursing intervention. ∗ indicates that there is a difference between the two groups.

Questionnaires were used to investigate the satisfaction of patients in both groups. The results showed that 37 (67.28%) patients were very satisfied, 12 (21.82%) patients were satisfied, 2 (3.63%) patients were not very satisfied, 0 (0.00%) patients were not satisfied, and the total satisfaction was 53 (96.37%) patients in SG. In CG, 20 (36.36%) patients were very satisfied, 24 (43.63%) patients were satisfied, 8 (14.55%) patients were not very satisfied, 3 (5.45%) patients were not satisfied, and the total satisfaction was 44 (80.00%) patients. In SG, patient's satisfaction was higher (*P* < 0.05) (Figure 3).

## 4. Discussion

Cerebral stroke is a common cardiovascular and cerebrovascular disease in clinic, which is mainly caused by abnormal blood circulation in the brain. It has the characteristics of high morbidity, disability, recurrence, and mortality and has a great impact on the physical and mental health of patients [12]. A survey shows that stroke has become the first cause of death and the leading cause of disability among Chinese adults in China [13]. DM is a metabolic disease with the highest incidence in the world at present. Hyperglycemia is most common, and its pathogenic principle is caused by abnormal insulin secretion or impaired biological effects [14]. Statistics show that the number of diabetic patients in the world was as high as 25.6% in 2015 [15]. DM is easy to cause various complications. Stroke is a common complication of DM, which is a severe complication [16], with a high mortality and morbidity rate. It develops in middle-aged and elderly people over 40 years and has an impact on multiple body functions of patients, requiring long-term drug use. Compliance and awareness of diseases directly affect patients' blood glucose status and mental health [17, 18]. At present, routine nursing intervention is often used in clinical treatment, but the effect is not obvious. Studies have shown that neurology nursing can improve this issue. Neurology nursing is the key nursing method of stroke complicated with DM at present. It can intervene the patient according to their different condition, simultaneously observe patients' blood sugar level, effectively improve their limb function, and accelerate the process of disease rehabilitation [19, 20]. The results showed that compared with the CG (6.93 ± 1.95, 12.79 ± 1.87, and 20.02 ± 5.68), the levels of fasting blood glucose, 2 h postprandial blood glucose, and urinary microalbumin in SG (6.01 ± 1.02, 11.18 ± 1.21, and 41.65 ± 6.07) were obviously better after treatment. Neurology nursing can observe patients' blood glucose level synchronously and control patients' blood glucose better (*P* < 0.05). After treatment, the proportion of patients in SG who basically recovered and had obvious improvement (38.18%, 49.09%) was higher than that in CG (20.00%, 30.91%), and the proportion of patients without treatment effect (1.81%) was obviously lower than that in CG (21.82%). Overall, the nursing effect of the SG (98.19%) after treatment was better than that of the CG (78.18%) (*P* < 0.05). There was no significant difference in SF-36 score between the two groups before nursing intervention (*P* > 0.05). After the intervention, the quality of life score of patients in SG (83.23 ± 5.87) was obviously higher than that in CG (72.14 ± 4.79) (*P* < 0.05). There was no significant difference in Morisky score between the two groups before nursing intervention (*P* > 0.05). After the nursing intervention, the patients in SG (38.21 ± 2.14, 42.85 ± 3.02, 41.21 ± 1.84, and 40.07 ± 3.78) were obviously higher than those in CG (28.25 ± 3.68, 30.02 ± 3.87, 31.65 ± 4.17, and 31.56 ± 4.25) in terms of controlling body weight, taking medicine according to doctor's advice, exercising properly, and controlling diet. These may be due to the fact that neurology nursing can accelerate the recovery of patients themselves and gradually recover the corresponding limb functions through limb rehabilitation training. Language exercise can repair the patients' language function and promote the recovery of expression ability. Basic nursing can restrain complications, thus accelerating the recovery process of patients and the speed of body recovery (*P* < 0.05). After nursing intervention, SAS and SDS scores of patients in SG (37.12 ± 2.01, 35.96 ± 2.34) were obviously lower than those of patients in CG (48.49 ± 3.65, 49.21 ± 3.17), and patients in SG were less affected by negative emotions. Neurological nursing also pays attention to psychological nursing. Nurses can communicate in time, explain patiently and enlighten patients, make patients understand more about their own condition, pay attention to their emotional changes, and mobilize their enthusiasm for treatment and cooperation, which is conducive to the recovery of patients' mental health (*P* < 0.05). Questionnaires were used to investigate the satisfaction of patients in both groups. The results showed that 37 (67.28%) patients were very satisfied, 12 (21.82%) patients were satisfied, 2 (3.63%) patients were not very satisfied, 0 (0.00%) patients were not satisfied, and the total satisfaction was 53 (96.37%) patients in SG. In CG, 20 (36.36%) patients were very satisfied, 24 (43.63%) patients were satisfied, 8 (14.55%) patients were not very satisfied, 3 (5.45%) patients were not satisfied, and the total satisfaction was 44 (80.00%) patients. The results showed that the neurology nursing was more meticulous and comprehensive, and the patient satisfaction in SG was higher (*P* < 0.05), which was consistent with the previous conclusions obtained by scholars.

To sum up, neurology nursing has better clinical effect on stroke patients with DM, has obvious rehabilitation effect, can improve patients' quality of life, and can reduce their negative emotions, which is worthy of extensive clinical promotion and application.

## Figures and Tables

**Table 1:** Comparison of baseline data between the two groups ($\bar{x}\pm s$).

Groups	Cases	Gender	Age	Average age	Medical history	Average course	Levels of blood glucose
SG	55	28/27	40-69	55.87 ± 5.64	2-8	5.13 ± 1.24	8.13 ± 2.45
CG	55	30/25	43-71	56.39 ± 5.82	2-8	5.51 ± 1.03	8.41 ± 2.05
t	—	—	—	0.476	—	1.748	0.620
P	—	—	—	0.635	—	0.083	0.537

**Table 2:** Comparison of blood sugar level between the two groups after treatment ($\bar{x}$±s).

Groups	Cases	FPG (mmol/L)	2hFPG (mmol/L)	ALB (mg/L)
SG	55	6.01 ± 1.02	11.18 ± 1.21	41.65 ± 6.07
CG	55	6.93 ± 1.95	12.79 ± 1.87	20.02 ± 5.68
*t*	—	3.100	5.361	19.296
*P*	—	0.002	<0.001	<0.001

**Table 3:** Comparison of nursing effects between the two groups after treatment (%).

Groups	Cases	Basic rehabilitation	Significant improvement	Improvement	Ineffectiveness	Total effective rate
SG	55	21 (38.18)	27 (49.09)	6 (10.91)	1 (1.81)	54 (98.19)
CG	55	11 (20.00)	17 (30.91)	15 (27.27)	12 (21.82)	43 (78.18)
x^2^	—	—	—	—	—	10.555
P	—	—	—	—	—	0.001

**Table 4:** Comparison of negative emotion scores between the two groups after nursing intervention ($\bar{x}\pm s$).

Groups	Cases	SAS	SDS
SG	55	37.12 ± 2.01	35.96 ± 2.34
CG	55	48.49 ± 3.65	49.21 ± 3.17
*t*	—	20.236	24.940
*P*	—	<0.001	<0.001

## Data Availability

The datasets used during the present study are available from the corresponding author upon reasonable request.
